# Supplementary material for: Transfer efficiency of organic carbon in marine sediments
Source: Nat Commun. 2022 Nov 26;13:7297. doi: 10.1038/s41467-022-35112-9 (PMC9701188; doi:10.1038/s41467-022-35112-9)
Supplement: Supplementary file 1 — Supplementary Information [file 41467_2022_35112_MOESM1_ESM.pdf]

Supplementary Information:

Transfer Efficiency of Organic Carbon in Marine Sediments

James A. Bradley[1,2*], Dominik Hülse[3,4*], Douglas E. LaRowe[5], Sandra Arndt[6]

[1]Queen Mary University of London, London, UK
[2]GFZ German Research Center for Geosciences, Potsdam, Germany
[3]University of California, Riverside, Riverside, CA, USA
[4]Max-Planck-Institute for Meteorology, Hamburg, Germany
[5]University of Southern California, Los Angeles, CA, USA
[6]Université Libre de Bruxelles, Brussels, Belgium

*J.A.B. and D.H. contributed equally to this work.

Corresponding author: James A. Bradley; jbradley.earth@gmail.com

This file includes:
Supplementary Discussion
Supplementary Figures 1 – 6
Supplementary Tables 1 – 2
Supplementary References

Supplementary Discussion

Diagenetic model evaluation. In general, continuum models of organic matter degradation such as the RCM used here are theoretically derived[1] and rest on a large body of organic matter depth profiles and rate measurements from a wide range of marine settings that show a decrease of organic matter reactivity with sediment depth and/or burial age. Over the past three decades, these have been successfully used to describe organic matter degradation dynamics across a range of contrasting depositional environments and over an extremely wide range of temporal scales from days[1,2] to hundreds of millions of years[3]. All of these diverse RCM applications were validated by comparing model output with comprehensive datasets of observed OM depth profiles, porewater depth profiles, rate depth profiles, isotopic signatures or authigenic mineral distributions (see ref.[4] for review) that confirm the ability of the RCM to reproduce observed diagenetic dynamics in marine sediments. Our specific model has been previously validated in ref.[5] by comparing our findings to previous regional to global empirical and modelling studies including refs.[6–11], and by ref.[12] using a global database of measured organic carbon concentration profiles, fluxes, and redox depths in sediment cores on the local and global scale. In addition, we compared our model output to five organic carbon (OC) profiles measured in sediment cores collected from different ocean depths and regions (Supplementary Table 2). For model simulations, we used our generic model setup and global parameters as described in the Methods (i.e. without tuning model parameters to local conditions). Results (Supplementary Figure 5) show that our approach captures the main features of observed OC concentration profiles across a range of different depositional environments and ocean depths without tuning model parameters. Importantly, due to significant local variability not captured by our global parameters, we do not expect our model to accurately simulate all local sediment characteristics (including OC profiles) globally.

Sensitivity Analysis. We carried out a global sensitivity analysis of four key model parameters: φ, ω, z_{bio} and D_b (see Methods). Our sensitivity analysis (Supplementary Figure 4) shows that output sensitivities inversely scale with sediment depth. Porosity and sedimentation rate are the most influential model parameters and have the most interaction with the other parameters at sediment depths greater than 50 cmbsf. The depth of the bioturbated layer has a large effect on OC burial rates in the upper-most sedimentary layers (i.e. 20 cmbsf) but its effect on OC burial decreases significantly in the deeper layers. The simulated OC burial rate is less sensitive to variations in the intensity of bioturbation (D_{bio}). While these results seemingly contradict the widely accepted notion that more bioturbation enhances OC degradation (e.g., ref.[13]), it is important to note that our global sensitivity analysis only accounts for the transport-related effect of bioturbation and does not include any direct interactions between bioturbation and OM reactivity (i.e., that OM is degraded faster in the oxic and well-mixed bioturbated zone). Additionally, the sensitivity indices calculated here are relative measures and can only be used to compare the influence of input parameters. The impact of a ±10% change of the two most influential model parameters (φ and ω) are shown in Figure 3 by the uncertainty envelopes (grey) on our modeled OC burial rates and T_{eff}'s. Supplementary Figure 6 shows the effect of these changes separated by input parameter.

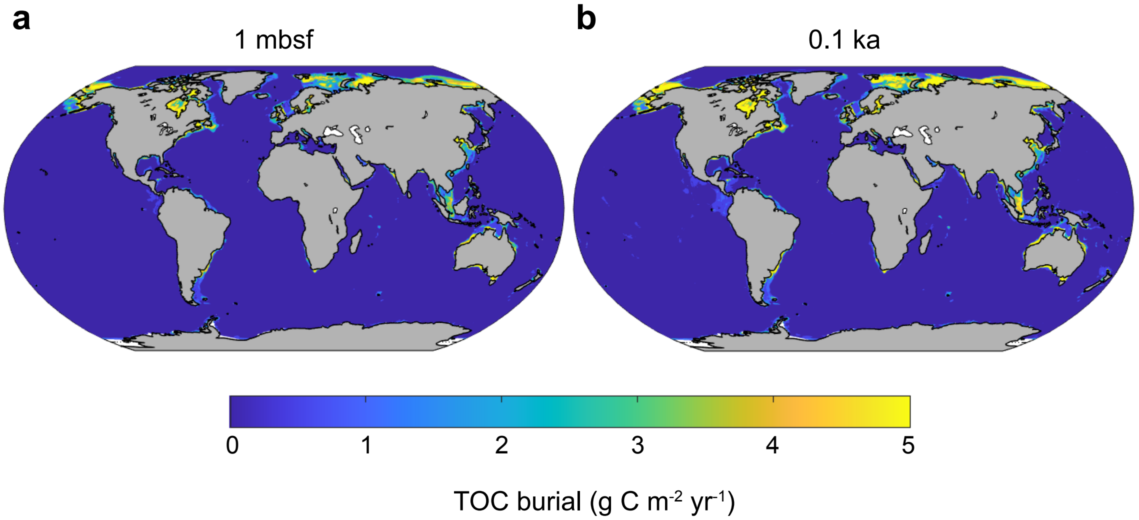

Supplementary Figure 1. Burial flux of organic carbon in global sediments. Total OC burial at a horizon of (a) sediment at a depth of 1 meter below the seafloor, and (b) sediment that was deposited 0.1 ka ago.

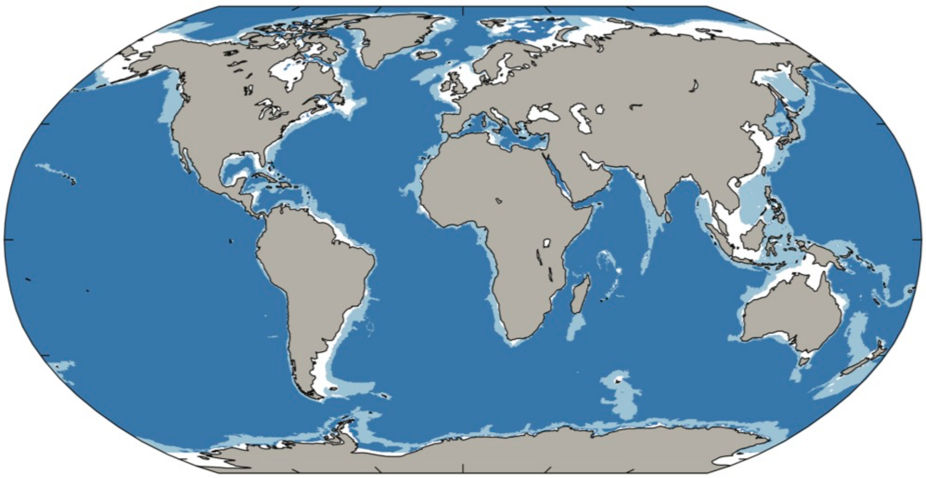

Supplementary Figure 2. Geographic distribution of major sediment domains. Shelf (white), margin (light blue) and abyss (dark blue) domains. Shelf environments roughly correspond to water depths <200 m, with the exception of the Antarctic region where shelf area corresponds to water depths <500 m; areas deeper than ~3500 m are taken to be abyssal plain; ocean floor covered by 500 to 3500 m water are referred to as margins.

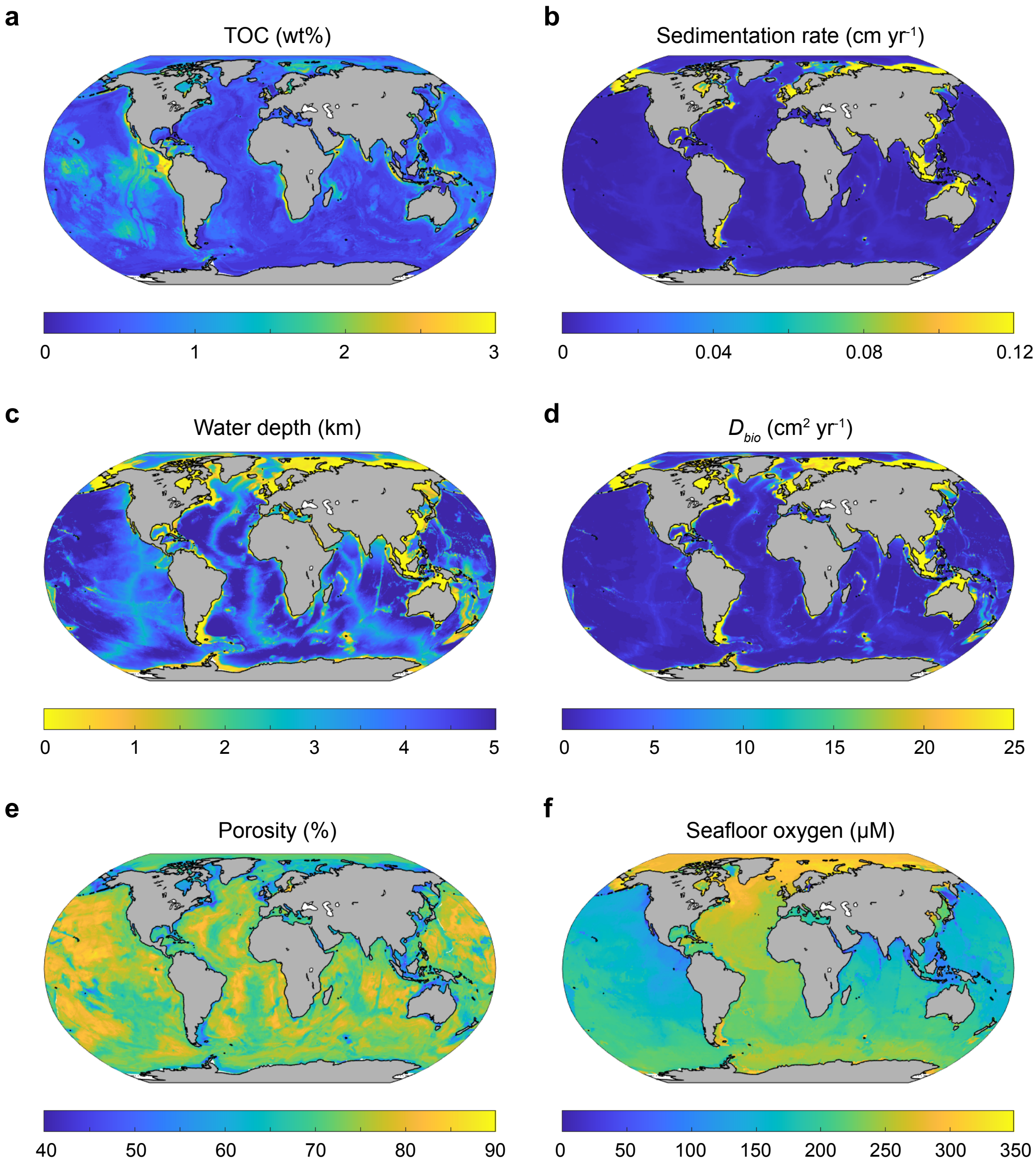

Supplementary Figure 3: Global maps of the nominal boundary conditions and parameter values used in this study.

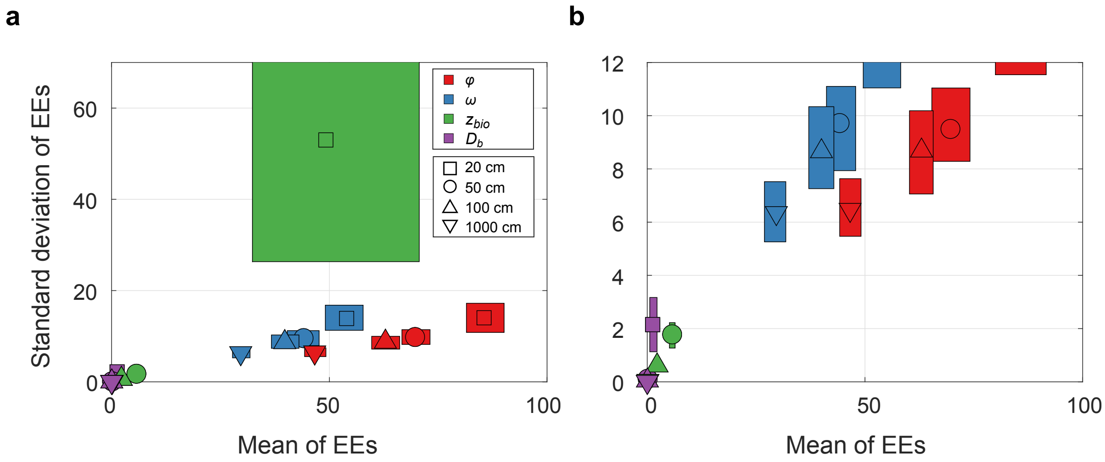

Supplementary Figure 4: Sensitivity analyses. Mean of Elementary Effects (EEs) versus their standard deviation for four key model parameters: φ representing the porosity, ω denoting the sedimentation rate, z_{bio} for the maximum depth of the bioturbated zone, and D_b for the bioturbation coefficient. Confidence bounds were derived via bootstrapping around the mean and standard deviation of the EEs. Sensitivities of OC burial rates to parameters φ, ω, z_{bio} and D_b were calculated at depths of 20, 50, 100, and 1000 cmbsf. Panel (b) shows the same as (a) but uses a different scale for the y-axis.

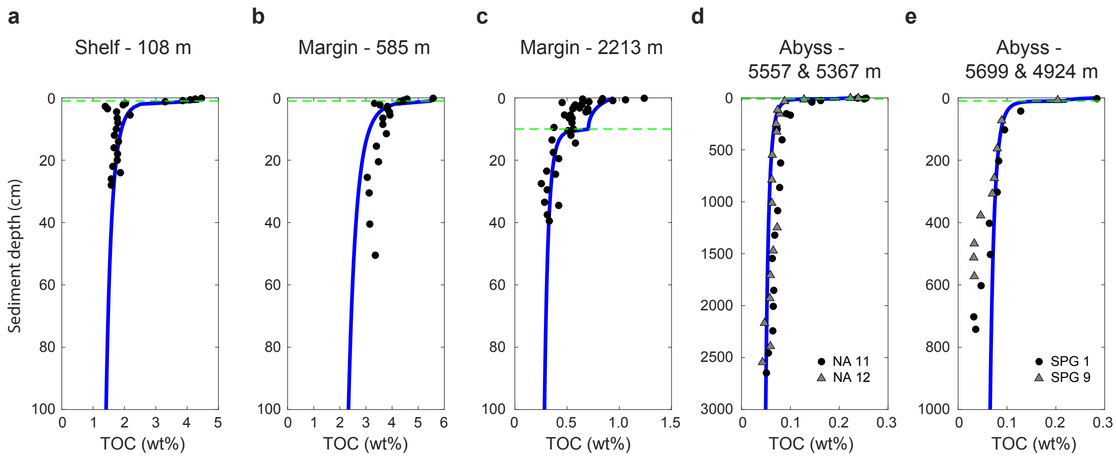

Supplementary Figure 5: Model-data comparison. Modelled (blue lines) and measured (shaded markers) OC concentration profiles for sediment cores. from: (a) the Iberian Shelf[14], (b) the Santa Barbara Margin[15], (c) the Iberian Margin[14], (d) the North Atlantic Abyss[6], and (e) the South Pacific Abyss[6]. zone and water depth). The panel headings indicated depositional environment and water depth. Further details on core measurements are provided in Supplementary Table 2. The green dashed lines indicate the depth of the bioturbation zone.

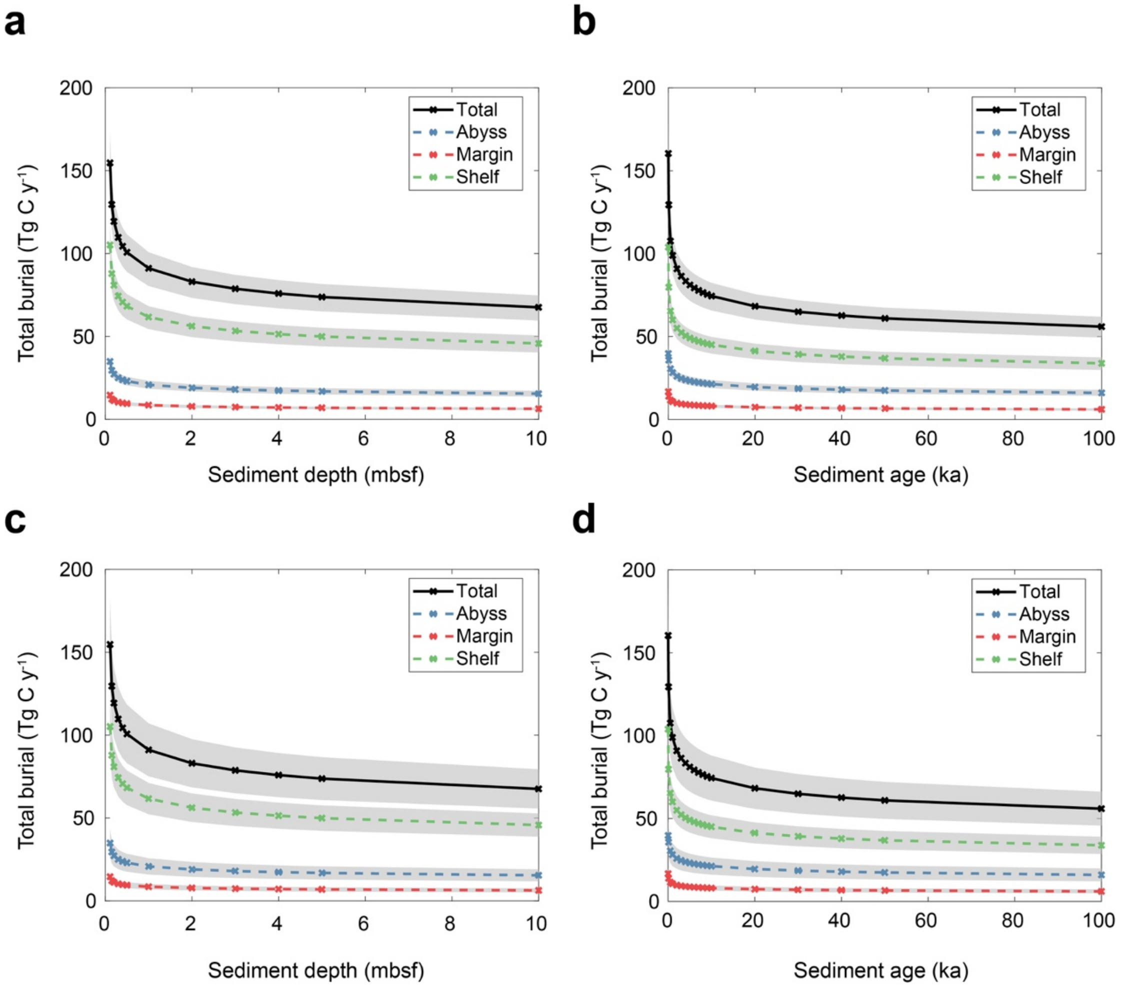

Supplementary Figure 6: Burial of organic carbon in marine sediments. OC burial in marine sediments according to (a,c) sediment age, and (b,d) sediment depth. Grey shading represents the variability in simulated OC burial arising from a change of ±10% in (a,b) the sedimentation rate, ω, and (c,d) porosity, φ.

Supplementary Table 1. Model estimates of organic carbon (OC) burial rate (in Pg C yr^{-1}) for the global ocean and the three major sediment domains at 0.11 mbsf. Lower estimates are calculated assuming a 10% increase in nominal sediment porosities and a 10% decrease in nominal sedimentation rates; upper estimates assume a 10% decrease in nominal porosities and a 10% increase in nominal sedimentation rates.

Area	Best estimate	Lower estimate	Upper estimate
Shelf	0.105	0.079	0.135
Margin	0.015	0.011	0.019
Abyss	0.035	0.024	0.048
Total	0.155	0.114	0.202

Supplementary Table 2. Boundary conditions for model validation simulations.

Area	Citation	Characteristics	Water depth (m)	D_b (cm^2 yr^{-1})	Sedimentation rate (cm kyr^{-1})	Porosity	OC_0 (wt%)	*a* value
Iberian Shelf	Ref.[14]	Thin bioturbated layer	108	27.260	107.10	0.599	4.44	0.1
Santa Barbara Margin	Ref.[15]	Anoxic bottom-water	585	17.620	10.50	0.695	5.50	10
Iberian Margin	Ref.[14]	Oxic bottom-water	2213	3.980	6.10	0.695	0.95	1
North Atlantic Abyss (NA 11 & 12)	Ref.[6]	Oxic bottom-water	5557 & 5367	0.200	0.26	0.850	0.26	20
South Pacific Abyss (SP 1 & 9)	Ref.[6]	Oxic bottom-water	5699 & 4924	0.233	0.339	0.850	0.29	20

Supplementary references

1. Boudreau, B. P. & Ruddick, B. R. On a reactive continuum representation of organic matter diagenesis. *American Journal of Science* **291**, 507–538 (1991).
2. Westrich, J. T. & Berner, R. A. The Role of Sedimentary Organic-Matter in Bacterial Sulfate Reduction - the G Model Tested. *Limnol. Oceanogr.* **29**, 236–249 (1984).
3. Arndt, S., Hetzel, A. & Brumsack, H. J. Evolution of organic matter degradation in Cretaceous black shales inferred from authigenic barite: A reaction-transport model. *Geochim. Cosmochim. Acta* **73**, 2000–2022 (2009).
4. Arndt, S. *et al.* Quantifying the degradation of organic matter in marine sediments: A review and synthesis. *Earth-Science Rev.* **123**, 53–86 (2013).
5. Bradley, J. A. *et al.* Widespread energy limitation to life in global subseafloor sediments. *Sci. Adv.* **6**, eaba0697 (2020).
6. Estes, E. R. *et al.* Persistent organic matter in oxic subseafloor sediment. *Nat. Geosci.* (2019). doi:10.1038/s41561-018-0291-5
7. Canfield, D., Kristensen, E. & Thamdrup, B. *Advances in Marine Biology: Aquatic Geomicrobiology*. (Academic Press, 2005).
8. Jørgensen, B. B. Mineralization of organic matter in the sea bed—the role of sulphate reduction. *Nature* **296**, 643–645 (1982).
9. Tromp, T. K., Van Cappellen, P. & Key, R. M. A global model for the early diagenesis of organic carbon and organic phosphorus in marine sediments. *Geochim. Cosmochim. Acta* **59**, 1259–1284 (1995).
10. Jørgensen, B. B. & Kasten, S. Sulfur Cycling and Methane Oxidation BT - Marine Geochemistry. in (eds. Schulz, H. D. & Zabel, M.) 271–309 (Springer Berlin Heidelberg, 2006). doi:10.1007/3-540-32144-6_8
11. Thullner, M., Dale, A. W. & Regnier, P. Global-scale quantification of mineralization pathways in marine sediments: A reaction-transport modeling approach. *Geochemistry Geophys. Geosystems* **10**, (2009).
12. Pika, P., Hülse, D. & Arndt, S. OMEN-SED(-RCM) (v1.1): A pseudo reactive continuum representation of organic matter degradation dynamics for OMEN-SED. *Geosci. Model Dev. Discuss.* **2021**, 1–36 (2021).
13. Aller, R. C. Bioturbation and remineralization of sedimentary organic matter: effects of redox oscillation. *Chem. Geol.* **114**, (1994).
14. Epping, E., Van Der Zee, C., Soetaert, K. & Helder, W. On the oxidation and burial of organic carbon in sediments of the Iberian Margin and Nazaré Canyon (NE Atlantic). *Progress in Oceanography* **52**, (2002).
15. Reimers, C. E., Ruttenberg, K. C., Canfield, D. E., Christiansen, M. B. & Martin, J. B. Porewater pH and authigenic phases formed in the uppermost sediments of the Santa Barbara Basin. *Geochim. Cosmochim. Acta* **60**, (1996).
